# A New Perspective on Job Lock

**DOI:** 10.1007/s11205-012-0072-2

**Published:** 2012-05-09

**Authors:** Anna Huysse-Gaytandjieva, Wim Groot, Milena Pavlova

**Affiliations:** 1 Department of Health Services Research, Faculty of Health, Medicine and Life Sciences, CAPHRI, Maastricht University Medical Center, Maastricht University, Maastricht, The Netherlands; 2 Topinstitute Evidence-Based Education Research (TIER), Maastricht University, Maastricht, The Netherlands; 3 Psychotherapie Praktijk Limburg, Valkenburgerweg 95, 6321 GC Wijlre, The Netherlands

**Keywords:** Job lock, Adaptation to job dissatisfaction, Job mobility, Self-esteem

## Abstract

This paper analyses the situation when employees fail to adapt to overall job dissatisfaction. By combining the existing knowledge in economics on job lock and in psychology on employees’ feeling of being ‘stuck’ at work, the paper explains why some employees fail to adapt when dissatisfied with their job. Thus, the paper aims to expand our understanding of why some employees are job locked or are ‘stuck’ at their work even though dissatisfied. Using the British household panel survey, the possibility of falling in a job-lock state is analyzed to outline a set of factors that explain why employees differ in the way they adjust to job dissatisfaction. We divide these factors into socio-demographic features, personality attributes, type of occupation, employment conditions, type of sector, and work-related contextual features. Based on results of probit regression analysis, we provide evidence that all these group of factors can jointly predict the state of job dissatisfaction, the absence of job turnover and job lock (being ‘stuck’ at job). Moreover, our results suggest that the adaptation to job dissatisfaction could be better understood if personality attributes (such as self-esteem) are included in the analysis. Thus, this study expands our understanding of how and why employees might feel ‘stuck’ at work and fall in a state of job lock.

## Introduction

Successful adaptation to feelings of dissatisfaction at work implies that an employee adjusts and consequently, job dissatisfaction remedies (Rosse and Miller 1984). This paper analyses the situation when an employee fails to adapt to overall job dissatisfaction. The inability to adapt to job dissatisfaction may have adverse effects on both the employee and the organization. It may lead to distress for the employee (Martin and Schermerhorn 1983; Rosse and Hulin 1985). Eventually, distress can cause physical and mental health problems (Rosse and Hulin 1985) that could result in low productivity for the organization (Chmiel 2000). Moreover, employees who stay dissatisfied may cause extra costs for the organization due to their negative attitude and withdrawal behavior that they may display (Hanisch 2002). Yet, much more is known about the reasons why employees leave the organization than about the situation of staying with the organization even though they remain dissatisfied (Mitchell et al. 2001).

Researchers use two terms to describe the situation of being dissatisfied with a job and not quitting. Economists use the term ‘job lock’ while psychologists refer to this situation as being ‘stuck’ at work. The term ‘job lock’ has not been clearly defined in the economic literature but it is mostly associated with employees who do not feel free to leave their jobs because of the limited portability of employer-related pension fund participation and/or health insurance, specifically in US (Adams 2004; Berger et al. 2004; Buchmueller and Valleta 1996; Dorsey 1995; Gilleskie and Lutz 2002; Gruber and Madrian 1993; Ippolito 1991; Kapur 1998; Madrian 1994; Monheit and Cooper 1994). Thus, job lock is treated by economists as dependent on external factors. More recent studies on job lock apply a difference-in-differences approach (Madrian 1994), which allows to distinguish the effect of employer-provided pension fund and/or health insurance from other (unobservable) factors that might lead to job lock. However, these studies have their shortcomings as well. Except for the additional demographic information, the analytical models applied in job lock studies are lacking personality factors that are essential for explaining behavior such as being dissatisfied with a job and not quitting (Miller and Rosse 2002).

At the same time, psychological research on employees’ feeling of being ‘stuck’ at work draws extensively on such personality factors to clarify what keeps employees hooked in their job even if they are dissatisfied. These factors refer to continued commitment (Allen and Meyer 1990), job investments (Farrell and Rusbult 1981), and job embeddedness (Mitchell et al. 2001). For example, employees who are dissatisfied with their job may choose to stay if: (1) they perceive a lack of alternatives (Allen and Meyer 1990); (2) they perceive that the costs of leaving the organization would be high, for example due to non-portable skills (Farrell and Rusbult 1981); (3) they are ‘stuck’ in the web of job embeddedness, e.g. due to the connection to others or the fit between the job with other aspects of life (Mitchell et al. 2001). Given these research outcomes in the field of psychology, it becomes apparent that apart from factors discussed in the economic literature, factors related to the personality of the employee, may also explain the decision not to move to another job even though dissatisfied. As Diener et al. (2009) stated, researchers are facing the challenge to discover the factors that control the adaptation process as well as better understand individual differences in adaptation. Knowing that adaptive behavior (those reducing the job dissatisfaction) is trainable (Frayne and Geringer 2000), we expect that knowledge about the adaptation process and the individual differences in it, would lead to taking more adequate actions in order to help employees to deal with adaptation issues on the workplace and to guide employees in efficiently addressing adaptation problems.

The aim of this paper is to combine the existing knowledge on job lock and employees’ feeling of being ‘stuck’ at work to identify a set of factors that explain why some employees fail to adapt when dissatisfied with their job. By combining economic and psychological perspectives, the paper expands our understanding of why some employees are job locked or are ‘stuck’ at their job even though dissatisfied. Thus, this paper provides a new perspective on job lock and being ‘stuck’ at work.

We use data from the British household panel survey (Taylor et al. 1999). The dataset provides us with longitudinal data that allows us to identify employees who have fallen in the state of job lock (i.e. ‘stuck’ with their job) since this requires data from several subsequent years. The dataset also allows comparing those in a job-lock situation with other employees. In meta-analysis on intention-behavior relationships, Webb and Sheeran (Webb and Sheeran 2006) recommend in future research to use more non-intentional routes to action. In this regard, the longitudinal data in this dataset allows us to apply the peak-end rule to derive the affective value of experience-related variable (such as self-esteem). The peak-end rule implies that the fluctuation in the evaluation of experiences lived across time can be represented by the most extreme (peak) and final (end) evaluation of these experiences (Kahneman et al. 1999).

The following two sections of this paper outline the main findings of previous research in economics and psychology relevant to our study. The subsequent sections present a description of the data and data analysis, the results of our analysis, and their discussion. At the end of the paper, conclusions for management and research are drawn.

## Job Lock, Being ‘Stuck’ at Work and Failure to Adapt to Job Dissatisfaction

Nowadays, workplaces require almost continuous adaptation by employees (Yeatts et al. 2000). Job dissatisfaction is an antecedent to job adaptation behavior at work (Hulin 1991; Judge and Hulin 1991). It is an unpleasant experience and as such it provides an incentive for the employee to find a way to deal with it (Hulin 1991; Rosse and Miller 2000). Adaptation theory implies that in response to job dissatisfaction, employees take decisions that result in various behavioral forms. Rosse and Miller (2000) for example, classify these behavioral forms into five categories: problem solving, exit, avoidance, equity-enhancing retaliation, and capitulation. Hulin (1991) provides a classification that comprises of two basic groups: psychological (e.g. lateness, hanging about on the job, and staying away from work responsibilities), and physical (e.g. absenteeism and job turnover). Hirschman (Hischman 1970) categorizes responses to job dissatisfaction in three categories: exit (i.e. job mobility), voice (comprising the attempts of the employee to change the work environment), and loyalty (passive behavior which implies waiting for things to change).

Despite the differences in these classifications, they all suggest that employees respond differently to job dissatisfaction. Some employees, although not all, search and find another job when they are dissatisfied (i.e. job turnover/mobility). The job turnover could be either internal (i.e. new job within the same organization) or external (i.e. new job within another organization). There is ample evidence that such behavior overall helps employees to increase their job satisfaction (Ehrenberg and Smith 2000; Greenberg and Baron 2003; Hom et al. 1992; Hom and Griffeth 1991; Krausz et al. 2000; Mobley 1977; Mobley et al. 1979; Porter 1973; Warters and Roach 1973). Other employees however, remain on the same job even though they are unhappy at work (Rosse and Saturay 2004). Those who remain on the same job, either adapt successfully and improve the level of their job satisfaction, or fail to adapt and stay dissatisfied (Rosse and Miller 1984). We refer to the latter situation as being in job lock (or being ‘stuck’ at work).

Thus, job lock (or being ‘stuck’ at work) is seen in this paper as the absence of internal or external job turnover even though the employee experiences continuing job dissatisfaction. The identification of factors that contribute to the occurrence of such situation is the core of this paper.

To define a plausible set of explanatory factors for our analysis, we reviewed the available empirical evidence related to possible determinants of job lock and being ‘stuck’ at work tested in previous studies. However, we also reviewed the evidence on possible determinants of job dissatisfaction and absence of job turnover since these two conditions are used to define the situation of job lock (or being ‘stuck’ at work). We considered evidence reported in both economics and psychology literature.

Similar to previous reviews (Locke and Latham 1990; March and Simon 1958; Porter 1973; Rosse and Miller 2000; Spector 1997), we also found in our review that there are two broad groups of factors that potentially determine the state of job dissatisfaction, absence of internal or external job turnover (job immobility), and/or job lock (or being ‘stuck’ at work). These are employee’s personal characteristics as well as work-related factors. Based on our literature review however, we also identified four specific sub-groups of factors that comprise these two broad groups:

- Socio-demographic features, including age, gender, marital status, education and health status (Ang et al. 1993; Bender and Heywood 2006; Booth and Francesconi 1999; Clark 1996, 1997; Groot and Verberne 1997; Kapur 1998; Royalty 1998; Weiss 1984).
- Personality attributes, such as the big five personality traits, self-esteem, locus of control, commitment, job embeddedness, and job investments (Alavi and Askaripur 2003; Allen and Meyer 1990; Barrick and Mount 1991; Farrell and Rusbult 1981; Griffeth and Hom 2004; Judge et al. 2001; Meyer and Allen 1991; Mitchell et al. 2001; Parkes 1994; Rosse and Miller 1984; Rosse and Noel 1996; Zlatanovic 2000).
- Employment conditions, for example, possibility for on-the-job training, insurance portability, employer-related pension plan, union membership, full- or part-time work (Disney and Emmerson 2002; Dorsey 1995; Elias 1994; Gruber and Madrian 1993; Hughes and Bozionelos 2007; Ippolito 1991; Krausz et al. 2000; Meng 1990).
- Work-related contextual features, for example unemployment rates in the region (Carsten and Spector 1987; Hulin et al. 1985; March and Simon 1958).

The first two sub-groups listed above concern employee’s personal characteristics and the last two sub-groups concern work-related factors. In addition to this, we consider that variations between different occupations in different sectors might exist with regard to how employees deal with job dissatisfaction. This suggests that the type of occupation and type of sector also need to be added as sub-groups of factors in our analysis. Thus, we define six sub-groups of factors that we expect to determine the state of job dissatisfaction, absence of internal or external job turnover, and/or job lock (or being ‘stuck’ at work). Although various combinations of these sub-groups of factors have been tested in previous research, none of the studies that we identified analyses the joint effect of all six-sub groups of factors on job dissatisfaction, absence of internal or external job turnover, and/or job lock (or being ‘stuck’ at work). We take this as an objective in our analysis.

From all these sub-groups, the group of personality attributes is especially essential for our analysis since we aim to combine economic and psychological perspectives to explain why some employees fall in the state of job lock (‘stuck’ with their job). In particular, as indicated by our review of literature, personality attributes have been taken into account in the area of psychology when studying the situation of being ‘stuck’ at work but not in the area of economics in conjunction with the group of work-related factors. We specifically focus on self-esteem as a key indicator of personality corresponding to the failure to adapt to job dissatisfaction. In the following section of the paper, the importance of self-esteem in relation to job dissatisfaction, absence of job turnover, and job lock is discussed.

## Self-Esteem as Determinant of Job Dissatisfaction, Job Immobility, and Job Lock

Judge and Hulin (1993) claim that there are four core self-evaluations that determine our disposition towards job satisfaction: self-esteem, general self-efficacy, locus of control, and neuroticism. Core self-evaluation is a single construct. Core-self-evaluations proved to be a better predictor of job satisfaction compared to the BIG five model (Judge and Bono 2001). Judge and Bono (2001) explicitly note that they consider “self-esteem to be the most fundamental manifestation of core self-evaluations as it represents the overall value that one places on oneself as a person”.

Job satisfaction (as well as job dissatisfaction) is one of the attitudes that the employee has towards work. It is related to other personal attitudes as well (Eagly and Chaiken 1993). Attitude theory distinguishes between affective (feelings), cognitive (beliefs), and behavioral (actions) attitudes. Attitudes originate from some basic system of values. As a consequence, there is a tendency towards internal consistency of attitudes. In addition, attitudes are perceived to play a role in adaptation by helping people to gain an understanding of the world. Thus, attitude theory argues that work-related attitudes are relatively stable in time and have an impact on behavior (Lecky 1969; Pervin 1975; Rogers 1951).

In the search for an answer to the question of what makes people happier, research shows that self-esteem is one of the main determinants of satisfaction (Sheldon et al. 2001). In general, self-esteem is defined as the attitude towards yourself. Correspondingly, people with positive self-esteem are more likely to report satisfaction with various dimensions in life, including job satisfaction (Judge and Bono 2001; Locke et al. 1996; Zlatanovic 2000). Rosenberg (Rosenberg 1979) perceives self-esteem as an attitude related to worthiness as a person and as essential to our behavior. Rosenberg (1979) argues that there are several ways in which our attitudes towards ourselves resemble our attitudes towards other things. The resemblances are related to the content of the attitude, direction (positive or negative), and intensity (how stable and long lasting it is). Thus, we can expect that job satisfaction and self-esteem would share the same content, direction, and intensity. In addition, self-esteem has a relation to the occurrence of dissatisfaction (Karanikola et al. 2007; Salmela-Aro and Nurmi 2007), adaptation (Graziano et al. 1997; Schweitzer et al. 1992), memory selectivity (Tafarodi et al. 2003) and difficulties with career decision-making (Saka and Gati 2007; Sorensen 2001).

Self-esteem is essential for explaining job turnover as well. Job turnover models have been influenced by decision theory, attitude theory, sociology and labor economics (Steel and Lounsbury 2009). Different turnover models paid attention to different personality variables but they all hypothesize that individual differences have an effect on job turnover (Hom and Griffeth 1995; March and Simon 1958; Mobley et al. 1979; Rosse and Miller 2000; Steers and Mowday 1981). However, there is no consensus about the approach to the integration of personality in models that try to analyze withdrawals or job turnover (Rosse and Noel 1996).

Allen and Meyer’s three component model of organizational commitment and turnover intentions (Meyer and Allen 1991) states that each component (affective, normative, continuance commitment) influences employee’s turnover intentions and behavior. The study of Jaros (1997) shows that the relationship between organizational commitment and turnover intentions may be more multifaceted than described in the Allen and Meyer model. The results of this study show that the three components of commitment have a different effect on turnover intentions. The employees’ affective commitment to the organization is the most important component of organizational commitment in predicting turnover intentions (Jaros 1997). Affective commitment and self-esteem motivate citizenship behaviors (Bergami and Bagozzi 2000; Tang and Gilbert 1994).

As Rosse and Miller (2000) state, how people deal with dissatisfaction depends on differences in personality and situation. Demographic characteristics have an effect on the way people adapt. Self-esteem also has a strong influence on individual behavior (Baumeister 1993). People with an above-average level of self-esteem can adjust better and are more socially effective (Jorgensen and Dusek 1990). The level of self-esteem may also be seen as a motivational trigger (Wood and Bandura 1989). Moreover, employees with low self-esteem more often use passive coping strategies in response to stress than those with high self-esteem (Kinicki and Latack 1990; Parker et al. 1997).

Thus, decisions people make are influenced by how they value themselves. Their self-value may be an answer to the discrepancies from the perfect rationality concept (Gao et al. 2004). Those with low self-esteem are more likely to attribute their unsatisfactory situation to themselves (Hirschy and Morris 2002) in order to maintain the way they look at themselves (Herzberg et al. 1964). In advance, turnover is a risky decision (Allen et al. 2007). Knowing that people with lower self-esteem are reluctant to take risks and engage in new activities (Sorensen 2001) and are more risk averse (Josephs et al. 1992), it could be expected that they are less inclined to use active coping, e.g. search for another job when dissatisfied with their current job.

Further, some people are maximizers and others are satisfiers (Schwartz et al. 2002). Maximizers care more about the outcomes of their decisions and the results of choices may convey information about themselves. Thus, being a maximizer with low self-esteem might bring even more stress to the person when facing an unsatisfactory situation in the decision process (Schwartz et al. 2002).

Given the considerations above, the inclusion of self-esteem in our study can be crucial for explaining why people respond in different ways to similar causes and levels of job dissatisfaction, and perform behavior that seem to be irrational in economic terms (not utility maximizing) such as staying in a job lock even though dissatisfied.

## Data and Variables

For our empirical analysis, we use the British household panel survey (BHPS). The BHPS is an annual longitudinal survey using a nationally representative sample of ca. 10,000 individuals living in 5,000 households in Great Britain. The sample includes only the adult members of the households. Individuals participating in the panel are interviewed in successive waves. Details about this survey can be found in (Taylor et al. 1999). We only use the data for the period from 1991 to 1996 when suitable questions on job satisfaction and job turnover were used. The sub-sample extracted for our study consists of all men and women in the panel who were employed for at least at two consecutive years (two survey waves) during the period mentioned above (excluding self-employed persons). Thus, the sub-sample includes 17,627 individuals. Due to missing data for job satisfaction, job turnover and/or self-esteem, data for 14,341 individuals are included in the analysis.

The availability of data in the BHPS is the primary criterion applied for the selection and operationalization of variables for our analysis. The following considerations are taken into account:

- *Job lock* (*being* ‘stuck’ *at work*) Previous research does not indicate how long it takes for employees to adapt and what is the time lag between different adaptation decisions. On the basis of the results of the research of Hanisch (2002) that shows that the average thinking about quitting is 1 year, we assume that if an employee continues to be dissatisfied with his/her job for 2 subsequent years and at the same time stays in the same job, the employee is in a state of job lock. The job lock variable is constructed as a dummy variable (0 = job satisfaction and/or internal or external job turnover in 1 or 2 subsequent years, i.e. not in job lock; 1 = job dissatisfaction and absence of internal or external job turnover in 2 subsequent years, i.e. in job lock).
- *Job dissatisfaction* The variable that presents job dissatisfaction is also constructed as a dummy based on the overall measurement of job satisfaction (0 = job satisfaction in 1 or 2 subsequent years; 1 = job dissatisfaction in 2 subsequent years). Job satisfaction is measured in the BHPS on a seven-point Likert scale. To transform it to a binary variable that indicates the state of job dissatisfaction, categories from ‘somewhat satisfied’ to ‘completely satisfied’ with work are collapsed to ‘satisfied’, and categories from ‘not satisfied at all’ to ‘neither satisfied nor dissatisfied’ are collapsed to ‘dissatisfied’. The category ‘neither satisfied nor dissatisfied’ is seen as indicative of not being all that satisfied with the job and hence, it is included in the ‘dissatisfied’ category. The reason for this is the existing evidence that respondents frequently give socially desirable answers to questions on job satisfaction (Arnold et al. 1985). Thus, they tend to over-present the level of their job satisfaction, also when stating that they are ‘neither satisfied nor dissatisfied’ with their job. We use a dummy to specify job dissatisfaction because a single-item measure of job satisfaction (respectively, job dissatisfaction) has proven to be an acceptable measurement instrument (Oshagbemi 1999; Van Saane et al. 2003; Wanous et al. 1997).
- *Absence of job turnover* (*job immobility*) The variable that presents the absence of job turnover considers both internal and external turnover. It is derived from the measurement of tenure. In the BHPS, tenure is expressed as the date when an employee has started working in his/her present position (i.e. not for the present employer), which includes both internal and external job turnover. Thus, the absence of job turnover is a dummy constructed as follows: 0 = internal or external job turnover when tenure is <2 years, and 1 = absence of internal or external job turnover when tenure is greater or equal to 2 years.

The main argument for constructing the above variables as dummies is that these variables are used to define the state of job dissatisfaction and the possible reactions to it in a similar fashion. Specifically, the above variables allow us to identify and study the following groups of employees:

- Employees who reported job dissatisfaction for 2 subsequent years (job dissatisfaction = 1).
- Employees who did not change their job for 2 subsequent years (absence of job turnover = 1).
- Employees who fell in the state of job lock (job dissatisfaction = 1, absence of job turnover = 1, job lock = 1).

Our objective is to explain the probability of falling in one of these states using a set of explanatory variables. For this purpose, the variables job dissatisfaction, absence of job turnover (internal or external) and job lock are taken as dependent variables. The explanatory variables that we use to explain variations in the dependent variables are presented in Table 1. The selection of the explanatory variables is based on the possible determinants of job dissatisfaction, absence of internal or external job turnover and job lock that we identify above (see the Sect. 2 based on a literature review, constrained by the availability in the BHPS. Thus, based on the results of the literature review, we divide the explanatory variables into two main groups (employee’s personal characteristics and work-related factors) and in six sub-groups: socio-demographic features, personality attributes, type of occupation, employment conditions, type of sector, and work-related contextual features. For each sub-group, we include relevant explanatory variables to represent that sub-group but depending on the data available in the BHPS. In addition to explanatory variables that represent the six sub-groups, we also add tenure as explanatory variable to indicate the exact strength of job dissatisfaction and job immobility.

**Table 1:** Descriptive statistics

Variable group	Variable	*N*	Range or coding	Frequency	Median	Mean	SD
Socio-demographic features (personal characteristics, explanatory variables)	Age	17,627	From 15 to 81		37	37.757	11.9729
	Gender	17,627	0 = woman 1 = man	9,039 8,588	0	0.4872	0.49985
	Health status	17,627	0 = bad 1 = good	726 16,901	1	0.9588	0.19873
	Marital status	17,627	0 = not married 1 = married	6,816 10,811	1	0.6133	0.48700
Personality attributes (personal characteristics, explanatory variables)	Min peak-end self-esteem	14,341	0 = high 1 = low	13,088 1,253	0	0.0874	0.28239
Type of occupation (work-related factors, explanatory variables)	Manager and administrators	17,627	0 = no 1 = yes	15,429 2,198	0	0.1247	0.33038
	Professional	17,627	0 = no 1 = yes	15,768 1,859	0	0.1055	0.30716
	Associate professional/technical	17,627	0 = no 1 = yes	15,736 1,891	0	0.1073	0.30948
	Clerical and secretarial	17,627	0 = no 1 = yes	14,081 3,546	0	0.2012	0.40089
	Craft	17,627	0 = no 1 = yes	15,799 1,828	0	0.1037	0.30489
	Personal and protective service	17,627	0 = no 1 = yes	15,689 1,938	0	0.1099	0.31283
	Sales	17,627	0 = no 1 = yes	16,367 1,260	0	0.0715	0.25763
	Plant and machine	17,627	0 = no 1 = yes	15,904 1,723	0	0.0977	0.29698
	Other occupations	17,627	0 = no 1 = yes	16,244 1,383	0	0.0785	0.26890
Employment conditions (work-related factors, explanatory variables)	Member of the trade unions	17,627	0 = no 1 = yes	15,245 2,382	0	0.1351	0.34188
	Promotion opportunities	17,627	0 = no 1 = yes	13,414 4,213	0	0.2390	0.42649
	Employer pension scheme	17,627	0 = no 1 = yes	13,925 3,702	0	0.2100	0.40733
	On-the-job training	17,627	0 = no 1 = yes	11,364 6,263	0	0.3553	0.47862
	Fulltime contract	17,627	0 = no 1 = yes	3,841 13,786	1	0.7821	0.41283
Type of sector (work-related factors, explanatory variables)	Private	17,627	0 = no 1 = yes	7,736 9,891	1	0.5611	0.49626
	Civil	17,627	0 = no 1 = yes	16,992 635	0	0.0360	0.18636
	Governmental	17,627	0 = no 1 = yes	15,561 2,066	0	0.1172	0.32168
	NHS or higher education	17,627	0 = no 1 = yes	16,509 1,118	0	0.0634	0.24373
	National industry	17,627	0 = no 1 = yes	17,442 185	0	0.0105	0.10191
	Non-profit	17,627	0 = no 1 = yes	17,198 429	0	0.0243	0.15410
	Army	17,627	0 = no 1 = yes	17,549 78	0	0.0044	0.06638
	Other sector	17,627	0 = no 1 = yes	7,736 9,891	1	0.5611	0.49626
Contextual features (work-related factors, explanatory variables)	Regional unemployment rate	17,627	From 6.40 to 13.70		9	9.4386	1.88590
Additional explanatory variable (indicators of the strength of dependent variables)	Tenure	17,627	From 0 to 51		3	5.4594	5.90355
Dependent variables	Job dissatisfaction	14,341	0 = satisfied 1 = not satisfied	16,252 1,375	0	0.0780	0.2682
	Absence of job turnover	14,341	0 = job turnover 1 = no job turnover	987 16,640	0	0.0560	0.2299
	Job lock/being ‘stuck’	14,341	0 = not in job lock 1 = in job lock	16,470 1,157	0	0.0656	0.2476

Although the coding of the explanatory variables presented in Table 1, is self-explicable, the coding of minimum peak-end self-esteem requires additional explanation. Minimum peak-end self-esteem is constructed using the peak-end rule. The peak-end rule is a formula that helps to estimate the global evaluation of a construct (i.e. self-esteem) during a given period (Fredrickson and Kahneman 1993). It states that the affective value of the construct at a certain moment is a simple average of the peak (the most extreme evaluation of the construct during the period) and the end (the evaluation of the construct near the end of the period). A more detailed explanation of peak-ends can be found in Kahneman et al. (1999).

We include minimum peak-end self-esteem due to the personality differences in memory selectivity (Tafarodi et al. 2003) that respondents may have experienced. The self-esteem variable, and thus, also the resulting minimum peak-end self-esteem, is derived from the answer to the question: “Have you recently been thinking of yourself as a worthless person?”. The response is measured on a Likert scale, self-reported measure of self-esteem, which has proven to be appropriate for large scale surveys and longitudinal studies (Robins et al. 2001). Inclusion of both positively and negatively worded items when measuring self-esteem, prove to bring systematic biases that restricts the measurement of the trait (Horan et al. 2003).

## Data Analysis

Our analysis consists of two steps. First, we test the effect of all explanatory variables shown in Table 1, on each of the three dependent variables separately: job dissatisfaction, absence of job turnover (internal and external), and being in job lock. Given the binary nature of the dependent variable, binary probit regression is used for the analysis (statistics software package STATA). Since the magnitude of the coefficients estimated in a binary regression, does not indicate the exact effect of an explanatory variable on the dependent binary variable (Long 1997), we also estimate the marginal effects of the explanatory variables. The marginal effects indicate the change in probability of observing code 1 in the dependent variable (i.e. being dissatisfied, not changing job, or being in job lock respectively) when the explanatory variable is increased with one unit. Marginal effects are also often called discrete changes in probability of being in a given state.

Second, due to the fact that we select only the employed people for our study (those who are employed and report their job satisfaction), this may led to a non-randomly selected sample. Thus, it might be that the sample over represents more satisfied workers. For this reason, we additionally perform a Heckman type two-step procedure in order to correct for non-randomly selected sample (Heckman 1979). Additionally, this procedure enables us to identify the determinants of turnover among the sample of dissatisfied employees and to analyze why some employees stay with their job even though they remain dissatisfied. First ‘being overall job dissatisfied’ in 2 subsequent years is modeled and then ‘not changing job despite being dissatisfied’ in 2 subsequent years’ is modeled. Occupation variable is chosen as an instrumental variable (variable that predicts job dissatisfaction but is not related to turnover among the dissatisfied employees in the binary regression analysis).

## Results from the Data Analysis

Table 1 presents descriptive statistics related to the explanatory variables used in our analysis. As can be seen from the table, the employees included in our analysis, are on average 38 years old. The sample is evenly divided among the gender groups. Most of the employees are married, their subjective health status is on average good and their min peak-end self-esteem is overall high. Employees are equally distributed among occupation groups and most of them work in the private sector. About one-third of the employees have promotion opportunities. Most employees work full time and only about one-third are part-time employees. Less than one-third of the employees in our analysis are members of a trade union, 64.3 % of them did not receive any job-related training and 21 % reported participation in an employee pension fund.

At the bottom of Table 1, the descriptive statistics related to the dependent variables is presented. The table indicates that 6.6 % of the employees in our study, are dissatisfied with their jobs but did not change their job, i.e. they are in a state of job lock.

Table 2 contains the results of the three binary probit regressions for being dissatisfied, not changing job, and being in job lock respectively. The table also presents the discrete changes in the probability of being in those states (i.e. the marginal effects). The estimates of these discrete changes in probability, indicate the effect of a one-unit increase in an explanatory variable on the probability of being dissatisfied, not changing job, and being in job lock respectively (as percentage).

**Table 2:** Results of binary probit regression

Explanatory variable	Dependent variables								
	Job dissatisfaction (0 = satisfied; 1 = not satisfied)			Immobility/absence of job turnover (0 = job turnover; 1 = no job turnover)			Job lock/being ‘stuck’ (0 = not in job lock; 1 = in job lock)		
	Coefficient	SE	Marginal effect (% points)	Coefficient	SE	Marginal effect (% points)	Coefficient	SE	Marginal effect (% points)
Socio-demographic features									
Age	−0.002	0.001	−1.39	−0.022**	0.003	−7.61	−0.004*	0.002	−2.40
Gender	0.403**	0.037	10.92	−0.017	0.057	−0.30	0.366**	0.039	9.29
Health status	−0.350**	0.066	−5.31	−0.046	0.127	−0.36	−0.350**	0.069	−5.06
Marital status	−0.024	0.034	−0.69	−0.077	0.058	−1.33	0.011	0.037	0.29
Personality attributes									
Min peak-end self-esteem	0.521**	0.046	11.18	0.207*	0.081	2.56	0.463 **	0.051	9.27
Type of occupation (reference category: clerical and secretarial)									
Manager and administrators	−0.334**	0.059	−5.67	−0.901	0.088	−1.02	−0.330**	0.064	−5.13
Professional	−0.096	0.059	−1.61	0.054	0.099	0.54	−0.064	0.064	−1.01
Associate professional/technical	−0.357**	0.064	−5.53	−0.042	0.094	−0.45	−0.320**	0.069	−4.62
Craft	−0.059	0.057	−1.03	0.014	0.107	0.13	−0.006	0.060	−0.10
Personal and protective service	−0.321**	0.067	−4.79	−0.114	0.100	−1.14	−0.290**	0.071	−4.08
Sales	−0.028	0.072	−0.39	0.036	0.107	0.33	−0.032	0.080	−0.41
Plant and machine	0.054	0.057	0.95	0.145	0.105	1.38	0.106	0.060	1.77
Other occupations	0.023	0.067	0.34	0.092	0.123	0.75	0.066	0.070	0.93
Employment conditions									
Fulltime contract	0.356**	0.053	6.73	0.014	0.077	0.18	0.416**	0.058	7.16
Employer pension scheme	0.126*	0.051	2.46	−0.327**	0.064	−5.15	0.176*	0.057	3.09
Member of the trade unions	0.158	0.056	2.81	−0.109	0.071	−1.58	0.081	0.063	1.26
On-the-job training	−0.045	0.034	−1.35	0.027	0.056	0.49	−0.029	0.034	−0.81
Promotion opportunities	−0.399**	0.049	−8.13	0.022	0.056	0.40	−0.629**	0.061	−10.55
Type of sector (reference category: private)									
Civil	0.283**	0.079	3.61	0.128	0.266	0.48	0.217*	0.083	2.63
Governmental	0.031	0.061	0.51	0.277	0.242	1.15	0.024	0.063	0.38
NHS or higher education	−0.079	0.081	−0.97	0.262	0.246	1.06	−0.110	0.085	−1.29
National industry	0.518**	0.119	4.36	0.418	0.348	1.20	0.501**	0.122	4.11
Non-profit	−0.346*	0.141	−2.45	0.348	0.270	1.29	−0.503*	0.167	−3.02
Army	0.268	0.216	1.24	0.624	0.374	1.67	0.256	0.228	1.12
Other sector	−0.009	0.040	−0.24	0.126	0.230	0.55	−0.117*	0.041	−2.81
Work-related contextual features									
Regional unemployment rate	0.007	0.008	0.82	−0.021	0.013	−1.56	0.005	0.009	0.57
Other explanatory variables									
Tenure	−0.001	0.003	−0.35	−2.306**	0.112	−23.26	0.012**	0.003	4.29
Other model characteristics									
Intercept	−1.388**	0.138		2.892**	0.357		−1.470**	0.143	
Observations	14,341			14,341			14,341		
Pseudo *R* ^2^	0.08			0.52			0.09		

* Significant at 5 % level

** Significant at 1 % level

Overall, the outcomes of this regression analysis show that among all employees, employees with low self-esteem are more likely to experience job dissatisfaction, to be immobile and to be in a state of job lock. In addition to this, employees working in specific occupations (i.e. manager and administrative, associate professional, personal and protective service,) less often report job dissatisfaction than other groups of employees and less often fall in a job-lock situation. Employees who are young, without employer pension scheme, and recently employed on the current job (tenure) significantly less often change their job than other employees. At the same time, being young, working long time on the same job and with employer pension scheme is associated with higher probability of being in job lock compared to all other employees. And finally, the same as for job dissatisfaction, being male with poor health, fulltime on the same job, with employment pension scheme, with no promotion opportunities working in civil, national sector significantly increases the probability that an employee is in a state of job lock, while the likelihood that the employee would experience job lock is lower for an employee who works in non profit sector.

Thus, when job dissatisfaction, job immobility and job lock are analyzed separately (taking all employees), the regression results for job lock show similarities with the results for job dissatisfaction but also some discrepancies when compared to the results for job immobility. It should be noted however, that in this step of the analysis, we compare those in a job lock situation to all other employees including both job-dissatisfied employees who change their job and job-satisfied employees who do not experience pre-conditions for job lock.

Table 3 presents the results of the two-step Heckman model, where we first select employees who are dissatisfied with their job and then we analyze the job mobility within this group. This way, we analyze the situation of job lock only among those dissatisfied with their job. The results of the second step of the Heckman model (i.e. for immobility/absence of job turnover) suggest that among employees who report job dissatisfaction for 2 subsequent years, those who are young, with low self-esteem, without an employer pension scheme, and working for a short time with the employer are more likely to remain immobile even though they are dissatisfied with their job (i.e. are in a job-lock situation).

**Table 3:** Heckman selection model of job dissatisfaction on job immobility controlling for occupation

Variables	Job dissatisfied (1) (0 = satisfied; 1 = not satisfied)		Immobility/absence of job turnover (2) (0 = job turnover; 1 = no job turnover)	
	Coefficient	SE	Coefficient	SE
Age	0.0068	0.008	−0.0221**	0.003
Gender	0.0633**	0.022	−0.0032	0.054
Health status	0.0252	0.052	−0.0412	0.127
Marital status	−0.0221	0.036	−0.0819	0.058
Min peak-end self-esteem	0.0535	0.079	0.2120**	0.081
Fulltime contract	0.0168	0.030	0.0053	0.074
Employer pension scheme	0.0718	0.124	−0.3406**	0.062
Member of the trade unions	0.0572	0.042	−0.0874	0.068
On-the-job training	−0.0237	0.022	0.0158	0.055
Promotion opportunities	−0.0398	0.022	0.0124	0.055
Civil	0.1540	0.132	0.1306	0.266
Governmental	−0.0111	0.158	0.3009	0.240
NHS or higher education	0.0010	0.149	0.2612	0.246
National industry	−0.1034	0.223	0.4583	0.347
Non-profit	−0.0083	0.176	0.3461	0.270
Army	−0.1118	0.276	0.6149	0.374
Other sector	0.0584	0.123	0.1484	0.229
Regional unemployment rate	0.0051	0.009	−0.0227	0.013
Tenure	0.2509	0.473	−2.3021**	0.1112
Manager and administrators	−0.0310	0.299		
Professional	−0.0106	0.034		
Associate professional/technical	−0.0412	0.031		
Craft	−0.0416	0.034		
Personal and protective service	−0.0066	0.033		
Sales	−0.0114	0.034		
Plant and machine	0.0131	0.033		
Other occupations	−0.0297	0.038		
Constant	−0.2129	0.315	8.22	2.8990** (0.353)
ρ	0.3667			
λ	−0.3213			
ρ	−0.8762			
Number of observations	14,341			
Censored observations	13,362			
Uncensored observations	979			

* Significant at the 5 % percent level

** Significant at the 1 % level

## Discussion of the Results

The results of the regression analysis allow us to study three groups of employees, namely employees who reported prolonged job dissatisfaction (in 2 subsequent years), employees who did not change their job for 2 subsequent years (incl. internal and external job turnover), and employees who were in the state of job lock. We discuss here the factors that we identify in our analysis as statistically significant determinants of these states.

### Socio-Demographic Features

From all socio-demographic features included in our analysis, age appears to be a significant determinant of the absence of job turnover and being in job lock (both when we consider all employees and when we consider only those dissatisfied with their job). Gender and health status appeared to be a significant determinants of job dissatisfaction and being in job lock (when we consider all employees). Marital status does not appear to be a significant determinant in any of the three regression models.

Our results support the findings of a meta-analysis which show strong relationship between job satisfaction and health (Faragher et al. 2005). This may be due to the current employment conditions which diminishes job satisfaction. Further, it can be expected that health problems diminish the quality of life and influences overall happiness. Our study confirms the results of the research of Rosse and Hulin (Rosse and Hulin 1985) which shows that those employees who are dissatisfied with their job and fail to adapt, thus who are in job lock experience more physical and mental health problems compared to those employees who adapt successfully.

We find that the increase of age results in a higher probability of job turnover and less chance of being in job lock, but this does not significantly increase the probability of reporting job dissatisfaction. The effect of age on job turnover can be explained by the fact that in our study we do not differentiate between internal and external mobility. From the other side, elderly employees are less likely to end up in job lock, which can be explained by the fact that the people adapt better when they grow older, they have had time to find a better match and have less need to change jobs.

We also find that being male significantly increases the chance of being dissatisfied and of being in a job lock (when we take into account all employees). Previous research has also shown that women tend to be more satisfied with their job than men (Clark 1997; Sousa-Poza 2000). Specifically, women are found to be more likely to quit their jobs (Booth and Francesconi 1999). An explanation may be that women are more able to refrain from work because they create their identity through interdependent relationships and view their jobs as less central in their lives (Singh et al. 2004). Alternatively, it may be more difficult for a man who is dissatisfied with his work to leave his job, given how important achievement and work are to men’s identity. It might be that men invest more in specific human capital (Booth and Francesconi 1999). As a consequence, for a man, leaving his job would probably lead to a pay decrease. Men also frequently have the responsibility of being the main income provider. This may also contribute to their higher probability of not changing jobs and ending up in a job lock.

### Personality Attributes—Minimum Peak-End Self-Esteem

Self-esteem (specifically, minimum peak-end self-esteem) was used in our analysis to represent the personality attributes that might contribute to falling in a state of job lock. According to our results, peak-end self-esteem shows a statistically significant effect on all three states (being dissatisfied with the job, not changing the job and being in a job lock) and in both steps of the analysis. The probability of reporting job dissatisfaction, staying on the same job and being in job lock increases when self-esteem becomes lower. Furthermore, the increases in minimum peak-end self-esteem increase the odds of staying on the same job. Thus, we can conclude that minimum peak-end self-esteem has an effect on job immobility, currently experienced job dissatisfaction and job lock.

Our study confirms that when we solely have information on the employees’ attitude towards him, we can draw conclusions about the content and direction of his/her attitude towards work (Alavi and Askaripur 2003). People with high and low self-esteem differ in their attribution style. Moreover, low self-esteem individuals perceive the “negative events as being more personally important than subjects with high in self-esteem” (Campbell et al. 1991). This may be an explanation why when experiencing prolonged job dissatisfaction low self-esteem employees who are dissatisfied with their job, choose more passive forms of adaptation and postpone their decision-making compared to those with high self-esteem.

We may expect that people with lower self-esteem invest less in human capital as a result of not foreseeing enough worthy future gains. As a consequence such employees are likely to have less satisfying jobs and fewer opportunities for change on the labour market (Ellis and Taylor 1983). On the other side, if we use the arguments of James (1890), people relate their self-worth to certain areas of their life and there is a strong relationship between differential importance and self-esteem for people with low self-esteem (Pelham and Swann 1989). Thus, it might be that those employees who are in job lock connect their self-worth to work and achievement and consequently are more fearful of failure in those areas.

### Type of Occupation

Type of occupation appears significant only for two states: job dissatisfaction and being in job lock (when we take into account all employees). Specifically employees working as managers and administrators, administrative professionals, personal and protective occupations are less likely to be dissatisfied with their job and in a job lock. One of the background principles of Holland’s theory (Holland 1985) is that the choice of occupation is an expression of personality. Thus, occupation is a strong predictor of job satisfaction (Near et al. 1978). Additionally, some occupations have a clear career ladder. Such occupations may provide more promotion opportunities, and the employees may therefore be more satisfied and less eager to move.

### Employment Conditions

Having a fulltime job, employment pension scheme and promotion opportunities are significant for the state of job dissatisfaction and being in job lock (when we consider all employees). This confirms the outcome of previous studies that fulltime workers are less satisfied (Sinclair et al. 1999). Besides, our study shows that fulltime working employees are more likely to stay dissatisfied and immobile. This can be explained by the importance of work to those who work fulltime. Additionally, it can be that commitment and job investments are higher for those working fulltime compared with part time workers. Further, availability of promotion opportunities is likely to make employees feel more satisfied and decreases the probability of being in a job lock.

The results of our study show that an employer-related pension scheme is statistically significant for reporting job dissatisfaction and being in job lock, as previous research also shows (Dorsey 1995). Moreover, we found that employees who have an employer-related pension scheme are significantly more likely to change their job (both when we take into account all employees and only those dissatisfied with their job). This contradicts previous research findings (Gustman et al. 1994) which reported that British employees who are covered by an employer-pension plan are found to be less mobile compared to those who are not covered. One explanation for our results might be that only when employees are dissatisfied, they remain immobile because of expected future gains and non-transferability of the pension. However, when we take into account all employees, a job lock situation is more often observed among those with a employer-related pension scheme.

### Type of Sector

The type of sector proves to be a strong predictor for the states of being dissatisfied and being in job lock when we take into account all employees. Employees in the non-profit sector are less likely to be dissatisfied and are less likely to be in job lock than in other sectors. People working in non-profit organizations are highly committed to what they do and highly involved in their jobs because they experience a high utility from their work (Bacchiega and Borzaga 2001, 2003). Consequently, such employees have less drive to quit.

### Work-Related Contextual Features

The only contextual feature included in the analysis, is the regional unemployment rate. It appears to be insignificant for all three states and in both steps of the analysis. This can be explained by the fact that the employees’ behavior might be influenced not by the actual job availability but by their perceptions of job availability (Van Vianen et al. 2003).

### Tenure

Tenure appears to be a significant predictor for job turnover and being in job lock. The longer the tenure the higher the chance that the employee would move. This can be explained with the fact that in our study we do not differentiate between internal and external mobility. And the longer the employee stays in a company the more chance he has to move internally. This also applies to the situation of job lock when we consider only employees who are job dissatisfied. Thus, when dissatisfied with their job, employees who work longer on the same job are more likely to change their job (either infernally or externally) than those who have been recently employed on the current job. However, when we take into account all employees, a job lock situation is more often observed among those who work longer on the same job.

## Conclusions

In our study, we have integrated economics and psychology knowledge on job turnover, job dissatisfaction and job lock (being ‘stuck’ at job). According to our definition of job lock, 6.6 % of the employees included in our analysis are in job lock, which indicates the relevance of the issue to vocational behavior.

The results of our analysis confirm that there are various factors that can explain why employees differ in the way they adjust to job dissatisfaction. We divide these factors into socio-demographic features, personality attributes, type of occupation, employment conditions, type of sector, and work-related contextual features, and provide evidence that all these factors can jointly predict the state of job dissatisfaction, the absence of job turnover and job lock (being ‘stuck’ at job). Moreover, our results suggest that the adaptation to job dissatisfaction could be better understood if personality attributes as self-esteem are included in the analysis. Thus, this study expands our understanding of how and why employees might feel ‘stuck’ at work and fall in a state of job lock.

This study has its limitations, which combined with the results suggest some ideas for future research. In our analyses we include just the employed population. We do not account for labour market exit (those who left the market due to low levels of satisfaction with their job as well as those unable to find employment). Thus, the sample is selective. The use of probit analysis gave us an opportunity to analyze the same individual and organizational characteristics longitudinally. However, due to the fact that all cases are suppressed in probit, there is no way to estimate the variance. Further, for future research it might be interesting to test what type of adaptation behavior employees, who are in job lock, use.

It should be noted however, that cultures have been differentiated as individualism versus collectivism (Hofstede 2001; Triandis et al. 1988) or embeddedness versus autonomy (Schwartz 2004). Due to the fact that the importance of self-esteem in collectivist and individualistic nations differs (Brown et al. 2009; Diener and Diener 1995), the results of this study should be carefully transferred to other cultural contexts.

Despite these limitations, the results of our study might be of interest to researchers studying job mobility, job satisfaction and adaptation as to human resource managers and vocational counselors. It is essential to be aware of the nature of self-esteem in order to be able to diagnose it and treat it properly (Sorensen 2001). Self-esteem moderates the relationship between role stressors and job satisfaction (Abraham 1999; Jex and Elacqua 1999; Nelson and LeRouge 2001). Employers may need to consider the employees’ preferences for certain levels of job stress while taking self-esteem in consideration (Nelson and LeRouge 2001). Even though there is still a debate, if self-esteem (seen as a personality trait) can be changed, some very promising results can be achieved when employees, human resource managers and vocational counselors pay attention to self-esteem issues. Moreover, there is increasing evidence that the brain constantly adapts, re-wires and re-balances itself (neuroplasticity) contingent on the experiences that someone is going through (Kolb and Gibb 2008). Thus, by consistently training to bypass the self-limiting beliefs and self-sabotage it is possible to establish healthy self-esteem. A powerful form of training are the sentence completion (Branden 1998) and Acceptance and Commitment therapy exercises (Hayes 2005; Luoma et al. 2007). Further, applying some advanced meridian therapies, which suggest that every limiting thought, upsetting feeling and memory is associated with disturbances of our body/mind energy system, can bring remarkably quick and thorough therapeutic effects (Connolly 2004; Dwoskin and Canfield 2007; Gallo 2002, 2005; McGraty et al. 1995, 2003).
